# Vascularized tunica vaginalis interposition flap for the treatment of recto-urethral fistulas

**DOI:** 10.4103/0970-1591.57914

**Published:** 2009

**Authors:** Rajendra Nerli, S. S. Amarkhed, M. B. Hiremath

**Affiliations:** Department of Urology, KLES Kidney Foundation, KLES Dr. Prabhakar Kore Hospital & MRC, Nehru Nagar, Belgaum 590 010, India

**Keywords:** Fistula, prostate, rectum, urethra

## Abstract

**Introduction:**

Recto-urethral fistula is a rare complication of pelvic surgery, trauma, or inflammation. The many techniques for repairing these fistulas vary in their success rates. We describe the use of vascularised tunica vaginalis flap interposition in the repair of a recto-urethral fistula.

**Materials and Methods:**

Three children who had developed rectourethral fistula following surgery for anorectal anomaly/Hirschsprungs disease underwent repair through the perineal approach and interposition of vascularised tunica vaginalis flap in between the rectum and the urethra.

**Results:**

Three patients, all males aged 6 to 14 years old, presented with passage of urine per rectum following surgery. Following repair of the recto-urethral fistula, there was no recurrence of fistula in the follow-up period ranging from 1 to 6 years.

**Conclusions:**

Vascularised tunica vaginalis flap interposition is a straight-forward technique that can result in successful fistula repair.

## INTRODUCTION

Recto-urethral fistula is an uncommon and often devastating complication of prostate or rectal surgery,[1–3] pelvic trauma,[4,5] radiation,[6,7] or an inflammatory process.[1,8] Most patients require definitive operative repair. The operative management of recto-urethral fistula remains controversial, with no single technique providing results that are successful enough to consider it the standard of care.[1,9–17] Because of the rarity of the condition, the series describing the success of various repairs are limited by the small number of patients. Despite the choice of the technique, there is a high rate of recurrent fistulas.

The two primary classes of techniques include local repairs (York-Mason, Transvaal, or Transperineal approach) and tissue interposition. The former has the benefits of low morbidity and low mortality, yet the latter has a well vascularised tissue flap into the area of compromised tissues to encourage healing. We report our experience in the management of recto-urethral fistula using vascularised tunica vaginalis flap interposition through the trans-perineal approach.

## MATERIALS AND METHODS

Children presenting with acquired recto-urethral fistula since 2000 formed the study group. The children were assessed for the initial cause of the fistula, symptoms at presentation, previous attempts at repair, presence of preoperative urinary or fecal diversion, length of time before tunica vaginalis flap repair, intraoperative or postoperative complications, length of follow-up, and evidence of fistula recurrence.

A total of 3 patients underwent vascularised tunica vaginalis flap interposition repair through the perineal approach during this period. The operation was performed with the patient in an extended lithotomy position. A transperineal incision was made extending up to the scrotum in an inverted U shape. The incision was carried down through the dermis and the subcutaneous tissue. This flap was then dissected free of the underlying tissues, carrying this dissection to the transperineal area outside of the sphincter complex. A space was created by dissecting between the sphincter complex/rectum and the urethra, until the fistula was identified. The tissue surrounding the fistula was mobilized to allow for a tension-free repair of the rectum and urethra. The rectal fistula opening was repaired with 3-0 interrupted vicryl sutures. The urethral fistula opening was repaired with 4-0 interrupted monofilament sutures (Polydioxanone, Ethicon, U.S.A.). Next, one of the testes along with its coverings was delivered into the wound from the scrotum. A tunica vaginalis flap was created so as to interpose (preferably the epithelial side facing the urethra) between the sutured urethra and the rectum. 2-0 absorbable sutures were placed into the tunica vaginalis flap and this flap was tied down interposing into the plane that was created between the rectum and the urethra. The skin and subcutaneous tissues were closed in layers keeping a small suction drain.

## RESULTS

All 3 patients who underwent vascularised tunica vaginalis flap repair during this period were males aged 6 to 14 years old. Two of these children had undergone pull-through surgery for an ano-rectal anomaly in the neonatal period and the third child had undergone extensive rectal surgery for Hirschsprung's disease. All 3 patients had symptoms of passing urine through the rectum, 1 child also had a history of pneumaturia and fecaluria. All 3 children presented to us 6 to 11 years after surgery. One child had a previous attempt at repair through the abdominal route but was unsuccessful. This child had a fecal diversion at the time of presentation to us. After these children were properly evaluated at our center including a retrograde urethrogram, it was discovered that all had a < 1 cm recto-urethral (proximal bulbar) fistula, not more than 4 cms from the anal verge. The wound was clean and no active infection was seen in any of them. A pre-repair fecal diversion via a colostomy was done 6 weeks prior to the repair of the rectourethral fistula.

All 3 patients underwent repair as described above. Postoperatively, 1 child had a perineal wound infection and partial wound dehiscence. This healed with daily dressing, sitz bath, and secondary suturing. A pericatheter urethrogram done 3 weeks after the repair revealed no leak in any of the patients. All 3 patients voided well after removal of the catheter 6 weeks after the repair. The follow-up period ranges from 1 to 6 years. None of the patients have rectal or urethral strictures and there was no recurrence of fistula in any of the patients.

## DISCUSSION

Acquired recto-urethral fistula can result from infection, trauma, cancer, or iatrogenic injury. Injury has been reported in patients undergoing prostatic, rectal, anal, and bladder surgery. Injuries detected intraoperatively can be immediately repaired, whereas unrecognised injuries of the rectum and urethra can result in recto-urethral fistulas. One study has reported the spontaneous closure of these fistulas in 3 out of 8 patients,[18] but persistence of symptoms after 3 to 6 months generally indicates a need for operative repair. Repair of recto-urethral fistulas can be problematic, thus illustrating the prevalence of so many different surgical options.

The York-Mason repair, a transsphincteric approach, is the most common technique used.[10,14] Renschler and Middleton[19] who reported the largest series of patients treated with the York-Mason repair, had success in 92% of the cases.

Trans-perineal repairs provide excellent access to the urethra and bladder neck and also allow for interposition of vascularised tissue. The placement of well vascularised tissue into the area of repair can enhance fistula healing. Transposition of the gracilis muscle has been described for reconstruction of large defects after pelvic reconstruction, even for patients who have had radiation therapy in the pelvis.[17]

Varma, *et al*.[20] described the use of a dartos muscle interposition flap for repair of a recto-urethral fistula. Similarly Gupta, *et al*.[21] reported on their experience and outcome of gracilis muscle interposition following repair of rectourethral fistula through the perineal route. A total of 15 patients were treated with a successful outcome for all. The successful use of tunica vaginalis as a second layer of neourethral coverage in hypospadias repair has been described in several cases.[22] The use of tunica vaginalis in urethral reconstruction was also reported by Sinha, *et al*.[23] for the first time. Kumar, *et al*.[24] describe the use of a pedicled tunica vaginalis flap to cover the anastomotic area after progressive elaborated perineal bulboprostatic anastomotic urethroplasty. We have used a vascularised tunica vaginalis flap interposition in repair of the recto-urethral fistula. This study involves a small number of patients, hence superiority of this procedure over other inter position techniques cannot be commented on. Similar to the dartos muscle, tunica vaginalis is an easily obtained, well-vascularised flap that can be interposed in between the rectum and the urethra. The harvest of this flap incurs minimal morbidity and does not require an additional incision. This procedure worked well for our patients.

## CONCLUSIONS

Vascularised tunica vaginalis flap interposition is a technically straight-forward technique that may be an excellent option for repair of recto-urethral fistulas.
